# Feasibility of mapping and cannulation of the porcine epicardial lymphatic system for sampling and decompression in heart failure research

**Published:** 2018-07-18

**Authors:** Benjamin Kappler, Dara R. Pabittel, Sjoerd van Tuijl, Marco Stijnen, Bas A.J.M. de Mol, Allard C. van der Wal

**Affiliations:** ^1^LifeTec Group B.V., Eindhoven, the Netherlands; ^2^Department of Cardiothoracic Surgery, Amsterdam, Academic Medical Center, the Netherlands; ^3^Department of Physiology, Hasanuddin University Faculty of Medicine, Makassar, Indonesia; ^4^Department of Pathology, Academic Medical Center, Amsterdam, the Netherlands

**Keywords:** porcine cardiac lymphatics, anatomy, edema, heart decompression, perioperative sampling

## Abstract

**Background and Aim:**

The cardiac lymphatic system drains excess fluid from the cardiac interstitium. Any impairment or dysfunction of the lymph structures can result in the accumulation of interstitial fluid, and may lead to edema and eventually cardiac dysfunction. Lymph originates directly from the interstitium and carries real-time information about the metabolic state of cells in specific regions of the heart. The detailed anatomy of the epicardial lymphatic system in individuals is broadly unknown. Generally, the epicardial lymphatic system is not taken into consideration during heart surgery. This study investigates the feasibility of detailed mapping and cannulation of the porcine epicardial lymphatic system for use in preservation of explanted hearts and heart failure studies in pigs and humans.

**Methods:**

The anatomy of the epicardial lymphatic systems of forty pig hearts was studied and documented. Using a 27 G needle, India ink was introduced directly into the epicardial lymphatic vessels in order to visualise them. Based on the anatomical findings thus obtained, two cannulation regions for the left and right principal trunks were identified. These regions were cannulated with a 26 G intravenous Venflon cannula-over-needle, and a Galeo Hydro Guide F014 wire was used to verify that the lumen was patent.

**Results:**

The main epicardial lymphatic collectors were found to follow the main coronary arteries. Most of the lymph vessels drained into the left ventricular trunk, which evacuates fluid from the left heart and also partially from the right heart. The right trunk was often found to drain into the left trunk anterior basally. Right heart drainage was highly variable compared to the left. In addition, the overall cannulation success rate of the selected cannulation sites was only 57%.

**Conslusions:**

Mapping of the porcine epicardial lymphatic anatomy is feasible. The right ventricular drainage system had a higher degree of variability than the left, and the right cardiac lymph system was found to be partially cleared through the left lymphatic trunk. To improve cannulation success rate, we proposed two sites for cannulation based on these findings and the use of Venflon cannulas (26 G) for cannulation and lymph collection. This method might be helpful for future studies that focus on biochemical sample analysis and decompression.

**Relevance for patients:**

Real-time biochemical assessment and decompression of lymph may contribute to the understanding of heart failure and eventually result in preventive measures. First its relevance should be established by additional research in both arrested and working porcine hearts. Imaging and mapping of the epicardial lymphatics may enable sampling and drainage and contribute to the prevention or treatment of heart failure. We envision that this approach may be considered in patients with a high risk of postoperative left and right heart failure during open-heart surgery.

## 1. Introduction

Despite its role in controlling cardiac fluid hemostasis and maintaining normal cardiac function, the cardiac lymphatic sys-tem (CLS) has been less studied than coronary vasculature. This lack of interest is probably due to the small size and complex network of lymph vessels. The CLS maintains cellular hemostasis by removing superabundant interstitial fluid from the cardiac cells. Thus, metabolic changes in cardiac muscle can be expected to be detected earlier in lymph than in venous blood. Consequently, analysis of the lymph could be used to identify early cellular metabolic changes.

Lymphatic obstructions sustained during surgery could result in interstitial fluid accumulation, which decreases the compli-ance capacity of the heart and leads to an increase in diffusion distance for nutrients and waste products. An increase in the amount of interstitial fluid results in cardiac edema followed by ischemia and a loss of cardiac function in the affected areas of the heart [1–3]. Depression of ventricular function with reduced ventricular contractility due to epicardial lymphatic obstruction has been documented [4]. Foldi et al. [5] observed myocar-dial damage and pathological changes on electrocardiogram af-ter lymphatic ligation, as well as an increase in serum transam-inase activity, similar to that seen in coronary occlusion. Fur-thermore, Lupinski [6] reported postoperative atrial fibrillation after unintentional damage to the lymphatic drainage of cardiac conductive tissue. In addition, post-transplantation cardiac graft failures have been associated with interruptions of lymphatic pathways and missing lymphatic connections [7,8]. Recent studies report the therapeutic approach of inducing lymphan-giogenesis by means of vascular endothelial growth factors. This is believed to promote healing after myocardial infarction thanks to the reduction of fluid accumulation and improved inflammatory cell clearance [9–11].

Analysis of lymph from cannulated vessels has demonstrated that metabolic changes in particular areas of the heart are dependent on the site from which the lymph is taken [4]. Hence, the anatomy of the epicardial lymphatic network must be fully understood to be able to cannulate lymph vessels draining the area of interest and to minimize interferences from other regions. There are far fewer studies on the anatomy and function of CLS than on coronary arteries and veins. However, several studies have described the lymphatic systems of pigs [6,12–14], and as pig anatomy has been found to be the most similar to that of the human, the porcine cardiac lymphatic system can be expected to most closely resemble the human cardiac lymphatic networks [14,15]. These studies describe the separate right and left lymph networks that drain the right and left heart, respectively, be-fore joining posterior of the aorta to form the common efferent trunk [12,13]. The common efferent trunk drains into the car-diac lymph node between aorta and trachea. However, Riquetl and Hidden [16] discovered that both trunks receive lymph from right ventricular regions and that the right trunk sometimes also runs anterior to the aorta to join the left trunk. From this, we in-ferred that the anatomy of epicardial lymphatic network has not yet been fully described. We further envision the importance of those anatomical variations which remain to be investigated in respect of possible drainage, sampling and decompression.

This study aims to illustrate and map the porcine epicardial lymphatic network in detail which will lead to a better under-standing of the network and its variations and potential applica-tions of this knowledge.

## 2. Materials and methods

### 2.1. Heart acquisition

Forty hearts were obtained from Dutch Landrace hybrid pigs of about 110 kg weight that had been slaughtered for human consumption. All slaughterhouse and laboratory protocols were compliant with EC regulations 1069/2009 regarding diagnosis and research of slaughterhouse animal material, administered by the Dutch Government (Dutch Ministry of Agriculture, Nature and Food Quality) and accepted by the related legal authorities of animal welfare (Food and Consumer Product Safety Authority).

### 2.2. Lymphatic staining

India ink solution (0.2% v/v, Royal Talens, Apeldoorn) was used for lymphatic visualization. The solution was injected directly into the epicardial lymphatic vessels with a 27 G needle.

### 2.3. Lymphatic cannulation

Intravenous Venflon cannulas (26 G) with a cannula-over-needle design were used to cannulate the left and right principal trunks. A Galeo Hydro Guide wire (F014, Biotronik, Berlin) was used to verify that the lumen was open. After the trunks were cannulated, India ink was injected apically. A cannulation was assumed to be successful when the apically injected ink entered the cannula.

## 3. Results

### 3.1. Anatomy

**Figure 1:**
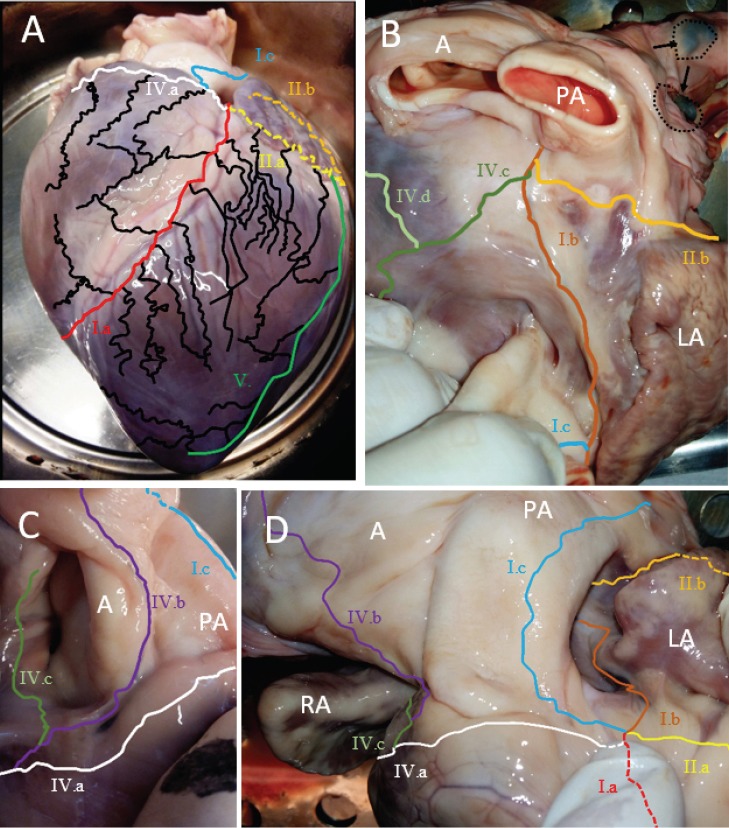
The principal routes of porcine epicardial lymphatic drainage at the anterior (A), plane view (B) and basal regions (C, D) of the heart. The Anterior interVentricular Trunk (AVT, Ia red line) runs from the apex region to the base of the heart; Ib brown line, normal route of the left principal trunk (LPT); Ic light blue line, alternative route of the LPT; IIa yellow line, normal route of the circumflex trunk (CXT) anteriorly; IIb orange line, alternative route of CXT posteriorly; IVa white line, right principal trunk (RPT) joining the AVT; IVb purple line, RPT along the aorta; IVc dark green line, RPT joins posteriorly the RPT; IVd light green, alternative course of the RPT along the vena cav; LA, left atrium; RA, right atrium; PA, pulmonary artery; A, aorta.

**Figure 2:**
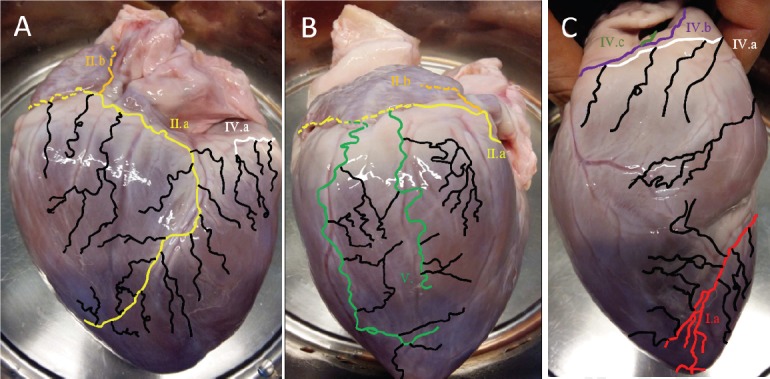
Inferoposterior and lateral views of the porcine epicardial lymphatics. A. The circumflex trunk (CXT, IIa yellow line) runs along the circumflex artery and drains the main inferoposterior and posterior lymph. Beside the CXT, the right principal trunk (RPT, IVa white line) arises and runs basal along the right ventricle. B. Two marginal trunks (MT, V green line) join the CXT laterally. C. The right lateral site with the possible routes of the RPT. IIa yellow line, anterior path of the circumflex trunk; IIb orange line, posterior path of the circumflex trunk; IVa right principal trunk,

**Figure 3:**
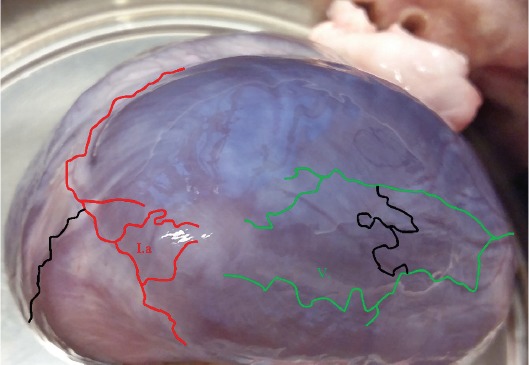
Apical view and origin of left ventricular drainage.The Anterior interVentricular Trunk (AVT, Ia red line) and the marginal trunks (MT, V green line) have their origin next to the apex of the heart.

Figure 1 shows the epicardial lymphatic network of the left anterior surface (Figure 1A) and the basal region of the heart (Figure 1B-D). In all 40 stained porcine hearts, the Anterior in-terVentricular Trunk (AVT, Ia red line in Figure 1A) always ap-peared to run next to the left anterior descending artery (LAD). The AVT mainly drained the lymph from the apex, Figures 2C and 3, and the medial regions of the anteroseptal right and left ventricle. At the base of the heart, in 97.5% of the cases (39 hearts) the circumflex trunk (CXT, IIa yellow line in Figure 1A) joined the AVT anteriorly. The two trunks formed the left prin-cipal trunk (LPT, Ib brown line in Figure 1B). However, in the remaining 2.5% (one heart), the CXT joined the AVT at the base of the heart posterior to the left atrium (IIb in orange line in Figure 1B). The LPT drained into the aortic lymph nodes (Figure 1B arrows and dotted circles). The LPT was found mainly in adi-pose tissue and it drained towards posterior aortic and pulmonary tissue. In one heart, an alternative route for the LPT was dis-covered which ran alongside the pulmonary artery (Figure 1D, Ic light blue).

The inferoposterior cardiac lymphatic networks showed fewer anatomical variations. In all hearts, the CXT was ob-served to follow the circumflex artery and drained large areas of the left and right inferoposterior ventricle (IIa, yellow line, Figure 2A). The apicolateral area of the hearts is drained by a variable number of marginal trunks (MT, V, green line in Figure 2B and 3) that go on to join the CXT. In all cases, the right principal trunk (RPT, IV a white line in Figure 2A) originated medioposteriorly, and close to the CXT. In all cases the RPT (IVa white in Figure 1D and Figure 2C) ran beneath the right atrium and was covered by adipose tissue. Where the RPT reached the anterolateral site of the right ventricle, considerable variation was observed in which four common paths could be found. The RPT (Figure 1D, IVa) was observed to frequently join (56%) the AVT anterobasally. However, in 23% and 21% of the cases, RPT ran via the aorta (IVb in Figure 1C and D) and beside the right atrium (IVc in Figure 1C and D), respectively. In 90% of the latter cases, the RPT joined the LPT posteriorly to drain lymph into the cardiac lymph nodes. In 4 hearts (10%), the RPT did not drain into the LPT and instead drained towards the V left marginal trunks. vena cava (Figure 1B, IVd, light green line). The anatomical findings of the epicardial lymphatic system were compared with results published in the literature (Table 1).

**Table 1:** Comments on published illustrations of the epicardial cardiac lymphatic system.

*Study aim*	*Species*	*Results*	*Comments*	*Reference*
*Investigation of endocardial, myocardial, and pericardial lymphatics as well as* *lymphatics of the valves*	• Dog, Pig, Human	• Dense network of subepicardial lymphatic capillaries joins larger vessels and accompanies coronary vessels	• Lymphatics of the atria could not be identified	[17]
*The role of the cardiac lymphatics in arterial fibrillation and cardiac surgery*	• Human	• LPT collects lymph from the AVT, AV node and bundle • AVT and CXT unite under the left atrial appendage to form the LPT • LPT runs along the left pulmonary artery • RPT collects lymph from the lateral and posterior walls of the right ventricle, right atrium and SA node • RPT runs posteriorly along the interventricular groove towards the base of the heart and ascending aorta • The RPT runs behind the ascending aorta or joins the AVT in front of the pulmonary artery to form the principal lymphatic channel or runs lengthwise along the superior vena cava	• Similar findings to the present study although variations of the RPT and LPT were not identified	[6]
*Cardiac lymphatic anatomy for cannulation purposes*		• Right and left drainage types were identified.	• Variations of the RPT and its merging with the LPT anteriorly were not described • Merging of the RPT and LPT behind the aorta was described	[4]
*Book about cardiac lymphatic system*		• Variations of the left site are less frequent than the right site • Identification of anterior mediastinal lymph nodes	• The mentioned anterior mediastinal lymph nodes were not found in the present study	[8]
*Distribution and variability*	• Human	• Lymphatic trunks followed the course of the coronary arterial system • Convergence of the right and left principal lymphatic trunks anterior to the pulmonary artery.	• In accordance with the present study	[18]
*Variations of the left atrium and ventricle*	• Human	• AVT passes over the pulmonary artery or posterior between the pulmonary artery and the left atrium • Indications that there are fewer variations of left lymphatic drainage than on the right	• The findings are consistent with present study	[19]
*Variations of the right cardiac drainage*	• Human	• RPT often joins the LPT anterior to the pulmonary artery • Alternative path in which RPT and LPT are separate from each other	• In accordance with the present study	[16]
*Epicardial lymphatics*	• Dog	• Epicardial lymphatics crossed blood vessels superficially • RPT passes to the right margin and to the left anterior interventricular to form the principal lymphatic trunk. • AVT joins the LPT, which originates at the CXT	• Different from our finding, the authors emphasized that the LPT originates as the CXT • Less frequent variations of the AVT	[20]
*Lymphatics of the cardiac conduction system*	• Human,Dog	• Conduction system drains either anteriorly into the RPT or posteriorly into the CXT • Parts of the conduction system are drained via the AVT	• The joining of right and left lymphatic trunks were not identified	[21]

Anterior interVentricular Trunk (AVT), left principal trunk (LPT), circumflex trunk (CXT), right principal trunk (RPT), sinoatrial node (SA node)

### 3.2. Cannulation

**Figure 4:**
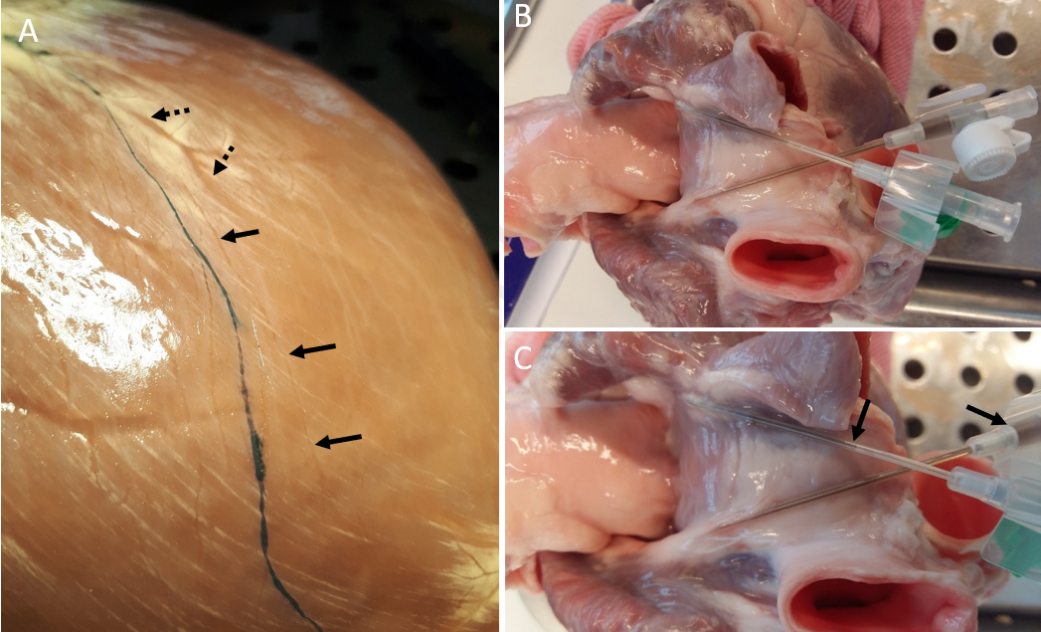
Staining and cannulation of the epicardial lymphatic system. A. Before cannulation, India ink (black line) was injected apically into the epicardial lymphatic collectors. Black arrows = unstained lymphatic vessel; dotted arrows = coronary artery. B. The left and right lymphatic trunks were cannulated with appropriately-sized intravenous Venflon cannulas. C. Close-up view of the cannulation site at the base of the heart and the India ink drainage through the cannulas (black fluid within the cannulas).

Due to the diversity of paths taken by the RPT, India ink had to be injected into the apical regions of lymphatic network to identify the RPT before cannulation with Venflon needles (Figure 4). The track of the LPT has less variation and could there-fore be identified without India ink staining. Nevertheless, after staining, cannulation of the trunks was successful, and the over-all success rate was 57% (23 of 40 hearts). In 22 of 40 (55%) hearts the RPT joined the LPT and formed a common trunk which was successfully cannulated in 16 of 22 cases (70%).

## 4. Discussion

We found a more complicated epicardial lymphatic network than has formerly been reported. Previously it was thought that there were only two principal lymphatic trunks draining the heart, namely the left and right principal trunks [6,8,12]. However, we found an LPT that drained lymph from the right and left ventricles and was more pronounced compared with the drainage on the right, which only transported fluid from the right heart. Additionally, the right-sided drainage system was observed to be highly diverse. These findings have not been well depicted in the literature and should be considered in epi-cardial lymphatic cannulation for lymph analysis and cardiac investigations. These findings also underline the previously described similarities of the human and porcine cardiac anatomy [16,22,23], particularly in their lymphatic systems. Additionally, the dense lymphatic meshwork was sufficiently stained with India ink, which has also been described in previous studies [24].

During direct epicardial lymphatic perfusion pressures and flows were not measured and could have led to a better perfu-sion and higher variability. However, the perfusion fluid fol-lows the course of the lowest resistance and therefore the course of the main lymphatic trunks towards the lymph nodes. Success-ful staining of cardiac lymph node was shown in Figure 1B and anatomical variations were only determined for the right drain-ing whereas the left was mostly invariable. AVT and CXT were found mostly at their predicted areas. These findings and the ev-idence that other studies described similar findings regarding the variability of the right drainage confirmed the suitability of the direct epicardial lymphatic perfusion.

In this study, we observed four different possible routes of the RPT. Primarily, the RPT joined the LPT at the basal an-terior region. In some cases, the RPT alternatively run along the aorta or between the aorta and the right atrium. In the latter cases, the RPT joined the LPT posteriorly or run along the vena cava. For this reason, in the event of lymph anomaly, the path of the right trunk should be taken into consideration during surgi-cal procedures involving the ascending aorta, such as proximalanastomosis during bypass grafting, antegrade cardioplegia per-fusion, aortic cannulation and aortic cross-clamping. The stand-ard route of the LPT towards the basal posterior site may differ; in some cases, the LPT drains along the pulmonary artery. Thus, its possible route along the artery should be considered in surgical procedures such as pulmonary transection (Fontan procedure) and pulmonary reconstruction (Rastelli procedure, repair of Fallot’s tetralogy and pulmonary stenosis/atresia) [6] in which swollen lymph structures are recognized.

Lymphatic vessels often overlapped the coronary arteries [13] and consequently increased the risk of injecting ink into the coronary system. The additional risk of ink particle aggregation could cause lymphatic obstruction and additional complications. To overcome the risk of particle aggregation, lymphazurin can be used. This is an alternative stain to India ink and is used to identify sentinel lymph nodes by injection into the surrounding lymph vessels.

Lymphazurin could be used to verify the different techniques for lymphatic cannulation described in the literature [13,25–27]. During the present study, a cannulation technique was developed by using Venflon cannulas with a cannula-over-needle design. This technique was reliable due to cannula flexibility. The can-nulas were introduced basally between the arteries and the atria as these locations were frequently the paths followed by the left and right principal trunks and could therefore be used for lym-phatic sampling and analysis. Due to the small structure of the epicardial lymphatics, cannulation is expected to be best con-ducted in arrested hearts, but cannulation in beating hearts is possible in experienced hands.

Given the similarity between the lymphatic systems of porcine and human hearts, the study described here enables the studying of preservation techniques and heart failure modalities in the ex vivo working model known as PhysioHeart [28]. The platform would offer the possibility to examine cannulation and drainage under a physiological beating status. Once the results look promising and reveal a potential patient application, the lymphatic system should be more systematically studied in fresh human cadaver hearts.

## 5. Conclusions

It proved feasible to illustrate the porcine epicardial lym-phatic anatomy in detail resulting in a map that enables iden-tification of sites for drainage of lymph. The variability of the right system remains a challenge for identifying an appropriate drainage site. However, the left lymphatic trunk also facilitates partial evacuation of the right cardiac lymph. The proposed can-nulation technique offers an acceptable cannulation success rate to obtain cardiac lymph for analysis of metabolic changes.

Epicardial lymphatic imaging and mapping may enable sam-pling and drainage which will potentially contribute to the pre-vention and treatment of heart failure. However, additional re-search in beating heart pig models is indicated to further estimate its relevance. Real-time sampling for biochemical assessment and lymph decompression may contribute to prevent or mitigate imminent heart failure during open-heart surgery.

## Conflict of interest disclosure

The authors have nothing to disclose.
